# Conceptualising household food insecurity in Northern Ireland: risk factors, implications for society and the economy, and recommendations for business and policy response

**DOI:** 10.1007/s43546-021-00070-9

**Published:** 2021-04-26

**Authors:** Emma Beacom, Sinéad Furey, Lynsey Hollywood, Paul Humphreys

**Affiliations:** 1 Department of Hospitality and Tourism Management, Ulster University Business School, Ulster University, Coleraine, UK; 2 Department of Hospitality and Tourism Management, Ulster University Business School, Ulster University, Belfast, UK; 3 Department of Management and Leadership, Ulster University Business School, Ulster University, Jordanstown, UK; 4 Present Address: Department of Food Business and Development, Cork University Business School, University College Cork, Cork, Ireland

**Keywords:** Food insecurity, Conceptual model, Stakeholder, Corporate social responsibility, Policy

## Abstract

Household food insecurity in developed nations has been identified as a significant public health concern. Although various research on the topic exists, such as contributors to food insecurity, and implications for individual physical and mental health outcomes; there is currently a lack of consideration as to how individual implications of food insecurity such as poor physical and mental health can consequently impact on business and the wider economy. In addition, there is a lack of conceptual literature related to food insecurity. Stakeholder interviews (*n* = 19) were conducted, and data were used to inform the conceptual model (risk factors, potential implications for individuals, the economy and business, and opportunities for business and policy response). The main suggested implications related to business and the economy were reduced contribution to the workforce and the economy, and increased cost pressures on the National Health Service. Business responses suggested included the inclusion of initiatives to address food insecurity in corporate social responsibility strategies, and further involvement of food businesses/retailers in redistributing surplus food. Policy responses suggested included policies relating to welfare, wages and work contracts, food redistribution incentives, sustainability, and community interventions in disadvantaged areas. The resulting model is unique in conceptualising food insecurity in the Northern Ireland context, with applicability to the UK and other developed nations.

## Introduction

Food insecurity, defined as “the lack of consistent access to adequate amounts of food” (Balistreri 2016, p. 373), has been identified as an increasing concern in the United Kingdom (UK) and other nations worldwide, presenting various implications for individuals’ physical and mental health, and ability to participate in societal norms (Garthwaite et al. 2015; Jessiman-Perreault and McIntyre 2017; O’Connell et al. 2019).

A variety of research on the topic exists, such as studies on predictors of food insecurity (e.g. Loopstra et al. 2019), how food insecurity is experienced (e.g. Heflin 2016), associated health outcomes (e.g. Ramsey et al. 2012), and studies appraising measurement approaches (e.g. Tanaka et al. 2020). There is however a lack of conceptual literature, particularly in the UK; a gap which this research seeks to address.

**Fig. 1:**
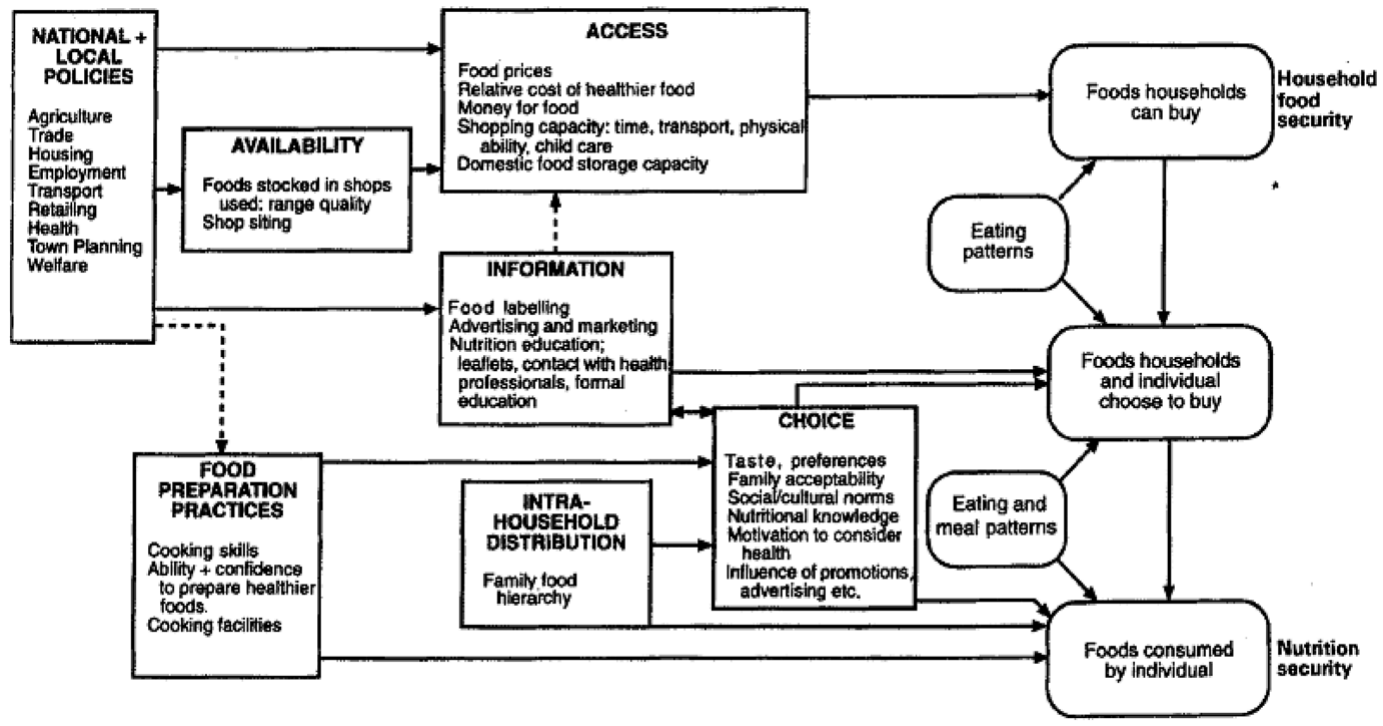
Dowler and Dobson’s (1997) framework of the determinants of food choice in the United Kingdom

Figure 1 presents a framework published by Dowler and Dobson (1997) which identifies the determinants of food (and nutrition) security in the UK. This model was informed by empirical research with low-income households in the UK which aimed to examine the relationship between nutrition and poverty (Dowler 1996). It focuses on both the macro-environment, and the household/individual level and displays various factors which can ultimately affect household food consumption. The model displays how various policies feed into issues of availability, access and information, and how these factors coupled with food preparation practices, household characteristics, and consumers’ choice preferences ultimately influence those foods which are bought and consumed. Dowler and Dobson therefore present a model of household food security, which is achieved when all the factors in the model align to provide consumers with the ability to access adequate foods of their choosing. Dowler and Dobson’s (1997) model does not identify individual characteristics which make households more susceptible to food insecurity, nor does it consider how knowledge of these characteristics, and how evidence of food insecurity in a population can influence national and local policy making. Further, the Dowler and Dobson (1997) model was created over 20 years ago, therefore this research will serve to update considerations in the model, particularly regarding policy changes. Dowler and Dobson’s model is deductive in nature as it considers general policies and their specific effects on household food consumption. Conversely, this study aims to provide a novel contribution to theory on this topic by creating an inductive model which, rather than using policy as a starting point and household food security as an ending point, considers household food insecurity as a starting point and its subsequent implications on business, the economy, and policy.

**Fig. 2:**
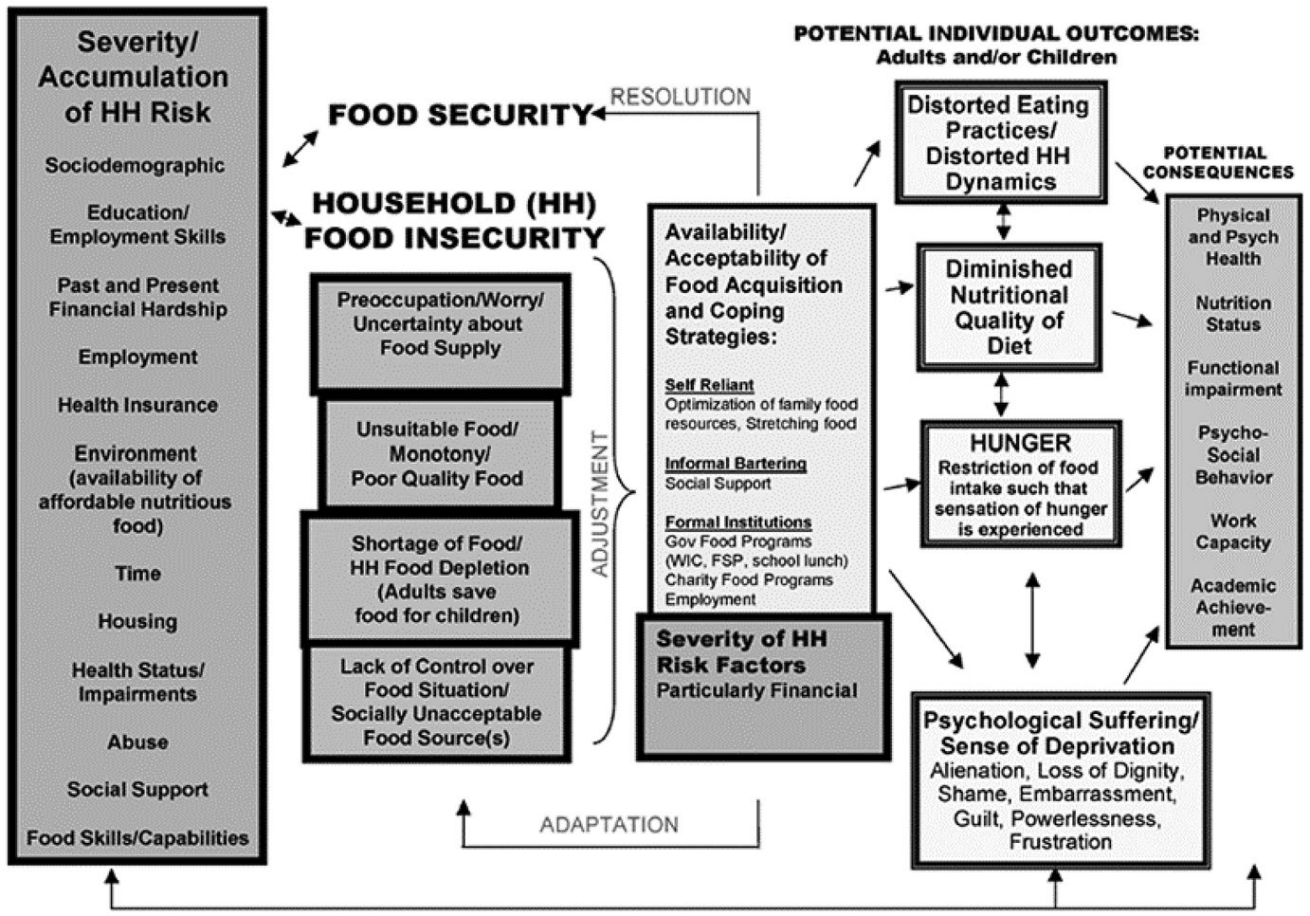
Alaimo’s (2005) conceptual model of food insecurity

Alaimo (2005) proposed a conceptual model of food insecurity components, determinants, outcomes and consequences (Fig. 2) in the USA. Unlike Dowler and Dobson’s (1997) model, Alaimo’s (2005) model includes various ‘household risk factors’, however, it was constructed over a decade ago, and in an American context. Further, this model was informed by the literature rather than empirical data, therefore this research will qualitatively examine stakeholders’ views on the household risk factors of food insecurity as proposed by Alaimo (2005), to adapt this model for the UK, and present-day contexts.

Alaimo’s (2005) model contains both short- and long-term individual consequences of food insecurity. There are some published studies which discuss the consequences of food insecurity for individuals (e.g. Ashiabi 2005; Ramsey et al. 2012), but there exists a gap in the literature as to the consequences of food insecurity for the economy and business (Humphrey et al. 2014). Some businesses have got involved in responding to the issue of food insecurity (e.g. through donations to food banks) as part of their corporate social responsibility (CSR) strategy or simply goodwill. However to date, there is limited consideration in the literature as to whether food insecurity can impact on business, or how implementing a measure and subsequently tackling and reducing the problem could benefit business. Further, responding to food insecurity has potential cost benefits for the economy; however these have not been commonly discussed in the literature. This research will seek to address this gap by qualitatively examining stakeholders’ perceptions as to the implications for business/the economy of food insecurity.

Among the two conceptual models discussed above, certain gaps were identified. Both models (Dowler and Dobson 1997; Alaimo 2005) included, to some extent, factors which could increase vulnerability to food insecurity, however a gap was identified regarding whether these risk factors are still considered relevant in the present-day context, and whether the risk factors identified in the USA (Alaimo 2005) are also relevant in NI/the UK. Although individual implications are presented (Alaimo 2005), there is no consideration as to the wider macroeconomic implications of food insecurity. Further, although Dowler and Dobson (1997) list policies which can impact upon food insecurity, there remained a gap regarding response to food insecurity from both policy (governmental) and business perspectives.

This study therefore aimed to address gaps in knowledge by conducting qualitative research to inform a conceptual model of food insecurity in NI, with applicability to the UK and other developed nations. Research objectives for this study were (1) to examine the household risk factors associated with food insecurity; (2) to consider implications of food insecurity for individuals; (3) to consider implications of food insecurity for business; and (4) to identify opportunities for policy/government response. Proposed elements for inclusion in this model therefore centred on these four objectives, highlighted formerly in italics.

## Methods

This research used a qualitative approach (stakeholder interviews) to test elements of both the Dowler and Dobson (1997) and Alaimo (2005) models to address prior identified gaps. The research sought to examine the ‘severity/accumulation of household risk’, ‘potential individual outcomes’ and ‘potential consequences’ sections of the Alaimo (2005) model. Further, as discussed previously, implications of food insecurity are generally discussed at the individual level rather than at the business or economy level, therefore this research sought to address this gap by examining potential implications of food insecurity on business/potential benefits for business if food insecurity was measured and addressed. Regarding the Dowler and Dobson (1997) model, only the ‘national and local policies’ component of the model was explicitly tested, in that stakeholders were asked their view as to policies upon which food insecurity could impact. Although the other sections of Dowler and Dobson’s (1997) model were of interest, and it was inevitable that some elements of these were discussed, greatest attention was afforded to the policy section to inform adaptation of this section in the resultant proposed conceptual model.

### Sample

A diverse range of stakeholders (*n* = 19) from Northern Ireland including consumer representatives (*n* = 5), community practitioners (*n* = 4), policymakers/policy officer (*n* = 3), political representatives (*n* = 2), local council representatives (*n* = 2), academics (*n* = 2), and a public health representative (*n* = 1) were interviewed. This sample was purposively chosen based on their work remit or interests directly or indirectly involving food insecurity. It was important that participants had the relevant knowledge/experience to speak authoritatively about the issues under investigation, to achieve the objectives of the research. Prospective participants with a range of relevant knowledge/experience were therefore selected (e.g. experience constructing/authorising regional or local measures and surveys, knowledge and experience of policy formation, knowledge about food redistribution operations, and experience working with those in food insecurity in response organisations or in the community more generally). Selected participants were identified (some were previously known to the research team, others were not) and contacted via email to explain the purpose of the research and what their participation would involve. A total of 30 suitable participants were contacted and 19 of these correspondences progressed to interview. Participants were contacted on an ongoing basis between October 2017–May 2018 and interviews continued until it was believed an appropriate number of groups had been represented and data saturation had been reached, indicated by continuous repeated comments and themes arising from the interviews. Informed consent was provided by all participants.

### Interview format

Interviews lasted between 30 min and 1 h, and were conducted by the primary author. Interviews were semi-structured and followed an interview topic guide (Appendix 1) which was compiled following consultation of the academic and grey literature, and considering the aims of the research. Initial questions assessed participants’ knowledge of food insecurity/direct experience through their work remit, and how they would define food insecurity. Interviewees were shown the risk factors section of Alaimo’s (2005) model and asked to confirm or otherwise if these identified risk factors were relevant in the NI/UK context. They were then asked to identify any further relevant risk factors. Interviewees were then asked questions relating to other sections of the proposed conceptual model, relating to the perceived implications of food insecurity for individuals and business/the economy, and how business and policy (government) could or should respond.

### Data analysis

Interview transcripts were read and re-read to achieve data immersion, then uploaded to qualitative analysis software NVivo v.12 and coded according to predetermined and emerging codes. Data were then deductively analysed by arranging relevant codes into the predetermined categories of the model. As recommended by Roller and Lavrakas (2015), these categories were then examined to determine if they should be further reduced into sub-categories. Analysis was conducted by the primary author, and codes and categories were checked by two additional researchers to increase the validity and reliability of results. Data within categories/sub-categories were then used to inform the resultant model.

## Results

**Fig. 3:**
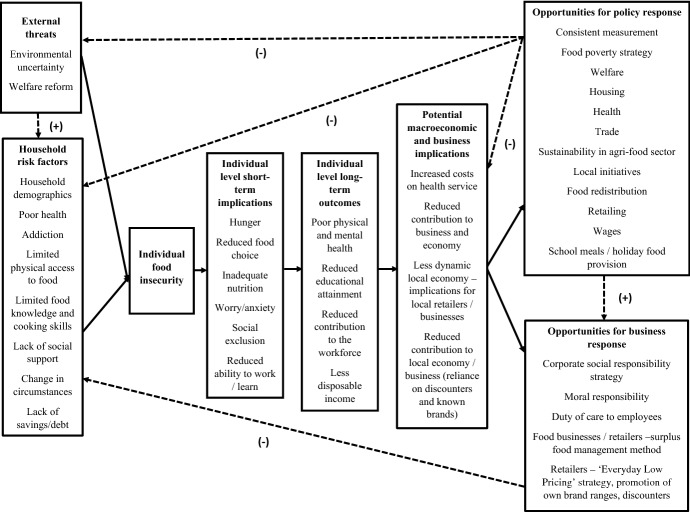
A conceptual model* of food insecurity risk factors, implications and opportunities for response. *Solid arrows represent the hypothesised positive (causal) relationships between the preceding and proceeding factor, dashed arrows represent the potential feedback (−) or feedforward (+) effect of the preceding factor on the proceeding factor

Prior identified categories for the model according to previous conceptual models and gaps in the literature were household risk factors; implications of food insecurity for individuals; implications of food insecurity for business; and opportunities for policy/government response. Results found that in addition to household risk factors, stakeholders also mentioned external contributors as impacting on susceptibility to food insecurity. Further, stakeholders cited both short- and long- term implications for individuals, and as well as discussing implications for business/the economy, they identified opportunities for business response. Therefore, the results from the study were themed into the following categories: household risk factors, external threats, individual level short-term implications, individual level long-term outcomes, potential macroeconomic and business implications, opportunities for policy response, and opportunities for business response. The resultant conceptual model is presented in Fig. 3. As discussed in the introduction, elements of this model were adapted from Dowler and Dobson’s (1997) and Alaimo’s (2005) conceptual frameworks. Results relating to the various components of the model are presented hereafter.

### Household risk factors

Stakeholders generally agreed that the household risk factors identified in the Alaimo (2005) model were relevant in the UK, aside from health insurance which a number of stakeholders (*n* = 10) noted as not relevant:

“It’s reasonably comprehensive…I can’t see anything that is glaringly missing.” (Public health representative)

A number of household risk factors were discussed by stakeholders (household demographics; poor health; addiction; limited physical access to food; limited food knowledge and cooking skills; lack of social support; change in circumstances; lack of savings/debt), as cited in the model. Two themes related to risk factors were discussed: micro-level and individual level contributors, and macro-level and economic-level contributors. Structural factors were most commonly cited as contributing to food poverty, followed by individual and political factors. Full findings and discussion related to the household risk factors of food insecurity as identified by stakeholders in this study are presented in a previous paper by the authors (Beacom et al. 2021).

### External threats

Certain stakeholders (*n* = 4) discussed environmental uncertainty in the form of Brexit, and the implications of leaving the EU on the food system in NI and food prices. Stakeholders considered how increasing food prices would present as a significant concern for consumers experiencing, or at risk of food insecurity, in further reducing the amount these consumers have available to spend on food, or resulting in them further squeezing their budgets to reduce spend on other essentials such as fuel.

A number (*n* = 8) also discussed welfare reform as an external factor outside of consumers’ control which could affect their amount of disposable income and therefore potentially increase susceptibility to food insecurity:

“Research has shown although [welfare reform] hasn’t really hit home here 100%, people waiting for their benefits - that can impact their diet and they would be even more likely to need emergency food provision.” (Community practitioner)

### Individual level short-term implications

As well as the immediate physical and mental consequences of food insecurity such as hunger, anxiety/worry, reduced ability to work or learn, and reduced nutritional intake, several participants spoke about the social aspects of experiencing food insecurity such as feeling excluded or different from peers. The social aspect of food insecurity was primarily discussed in relation to children, in that parents often want to provide certain items or experiences for children so that they are not seen as different from their peers. Reduced participation of adults in the workforce as a result of food insecurity was also considered, both from the perspective of people not participating in the workforce, and having a reduced ability to contribute productively:

“If someone is hungry then their productivity could be damaged [and] there could be more absenteeism because they get sick.” (Policy maker)

Certain interviewees commented how reduced income diminishes choice as there is a risk associated with buying food products that may not be eaten:

“[Finances] can reduce people’s choices in what they buy and choices to eat healthy.” (Consumer representative)

The risk associated with buying foods that may not be liked and eaten was discussed with particular regard to healthy foods. It was perceived that those who are struggling financially cannot afford to make choices like this as they need to ensure all food bought will be eaten, therefore they may instead buy cheaper, more filling foods, rather than allowing children to experiment with tasting different healthy snacks such as various fruits:

“I can afford to try things that I’ve never tried before and if I don’t like it it’s no loss, [those who are struggling financially] can’t.” (Public Health representative)

### Individual level long-term implications

Health (physical and mental) was a dominant theme discussed throughout (*n* = 15) as both a contributor to, and consequence of, food insecurity. Over half (*n* = 11) of stakeholders discussed the perception that healthy foods tend to be more expensive, or that unhealthy foods are often more filling and therefore more cost-effective, making it more difficult for those on low incomes/those who are food insecure to afford a healthy diet, which can subsequently lead to health problems:

“The worst things for me are the cheapest. It actually is much more expensive to eat healthily…and then you wonder why people in highly deprived areas have diabetes, obesity, the physical strain, then they have arthritis.” (Political representative)

Four stakeholders reflected on the connection between food insecurity and obesity, and one of these, from a health policy background, considered the links between obesity and subsequent long-term health problems such as cancers and heart disease. Of those stakeholders who discussed health, the majority (*n* = 12) referred to mental health, which was discussed as an implication of living in food insecurity.

It was considered by some (*n* = 6) that children from food insecure households who attend school hungry can have reduced concentration levels and therefore hindered learning. Some stakeholders (*n* = 3) discussed how this could consequently lower their educational attainment:

“[A] holiday hunger survey … indicated that children … [who were] fed healthy [and] regularly [over summer], maintained their level of education, those children who were surviving on poor diets when they came back, it took them a number of weeks to regain their education [attainment] because they hadn’t been nourished. So if we escalate that on further up life… then you are potentially getting graduates maybe with a lesser educational [attainment]. [Businesses are] reliant on the ability and the skills of [people], so in that aspect then yes absolutely [food insecurity can impact on business]”. (Local Council)

### Potential macroeconomic and business implications

Various economic and business implications were identified, such as the increased cost burden members of a society living in food insecurity contribute, as well as the potential loss to the economy through reduced contribution by those in such circumstances.

It was considered important to take a long-term view when considering the costs of food insecurity as responding to it now could ultimately save costs later:

“You have to look years down the line and see well what really is the cost to society, as well as individuals, by not addressing it.” (Consumer representative)

Four stakeholders considered the economic costs of members of a population experiencing food insecurity in terms of related healthcare costs on the health service:

“If somebody isn’t getting a good diet what’s going to happen is they’re going to get sick, or they’re going to get sick more quickly, and then the cost of that falls on the health service...” (Consumer representative)

Some stakeholders (*n* = 5) considered how households or individuals who are experiencing food insecurity will have limited disposable income, and therefore their contribution to business (and subsequently to the economy) will be reduced:

“If we can’t afford food, if we don’t buy it, or we don’t buy it in the same quantities, that’s going to impact the revenue stream, the profitability of our private sector business.” (Academic)

It was further discussed how those on limited incomes were considered more likely to shop in cheaper corporate stores rather than supporting the local economy by buying local produce:

“You’ve got people shopping in supermarkets like [anonymised] for their food and not affording local stuff, so what impact does food poverty have on the local agri-business?” (Consumer representative)

In addition, the growing consumer trend towards shopping at discounters and desire for value products was discussed as creating competition among retailers to lower prices and therefore reduce their profit margins:

“We know that consumers are shopping around more because money is tighter and food bills are a worry, and if the situation with food poverty were to increase then I imagine that that is even more of a lever really…[and retailers would have to] try to copy the discounters’ model.” (Consumer representative)

Enabling people economically would have presumably durable benefits for both the local and wider economies:

“Ultimately that puts more money in our consumers’ pockets, and we all have to eat, so that will help the local economy spread the wealth across food retailers in terms of consumers procuring food, businesses having to exist to supply it.” (Academic)

### Opportunities for policy response

Identified recommendations for policy and practice centred on the need for regular monitoring of food insecurity, and various responses which should be implemented at both policy making and community levels.

For the most part, stakeholders spoke generally about types of policies which food insecurity is related to, rather than naming specific policies. References to how food insecurity could impact on different themes of policy were indirect, but largely in agreement with those identified by Dowler and Dobson (1997). The only specific policy which was discussed was ‘Preventing and Addressing Overweight and Obesity in Northern Ireland 2012–2022: ‘A Fitter Future for All’ which references the need for a co-ordinated approach to address food insecurity. Although not referencing specific policies, other stakeholders also discussed how food insecurity could have implications for health policy. For example, one discussed how human rights policy frameworks mandate that adequate food is a basic human right and government have an obligation to meet this need if people cannot themselves, while two stakeholders discussed how food insecurity was linked to health and therefore there was a need for higher level strategic messaging and regulations related to consumption of adequate, healthy food for the population.

A majority of participants (*n* = 13) expressed that a clearly defined measure, and targets that could be monitored and over which government could be held to account were important:

“Government, clearly they’ll want to have something they can be held to account over.” (Academic)

The potential usefulness of a specific UK food insecurity strategy or policy was suggested by three participants:

“I definitely think if there was an indicator that would be so useful; and then a strategy following that to try and tackle food poverty would be amazing.” (Local council)

In the absence of a government strategy or agreed action concerning food insecurity, interviewees recognised that, beyond practical aid, there is a limited amount that those working on the ground can do to try and improve the situation of those in, or vulnerable to, food insecurity:

“We can’t march on food retailers and say we demand that you lower your cost. We can’t march to [the local Parliament building] and say we demand that you increase benefits. The only thing we really can do is practical interventions.” (Consumer representative)

Stakeholders discussed various examples of community level interventions such as breakfast clubs (*n* = 3), the Cook It programme (*n* = 3), and social supermarkets (*n* = 8), but recognised that the extent to which interventions such as these are implemented is dependent upon the amount of funding available.

Food insecurity was thought to impact on welfare policies in that tracking it alongside implementation of welfare reform could show whether such reforms are problematic for the food insecure. Further, as welfare is designed to help those in need, measuring the extent of food insecurity could lead to changes in welfare policies through evidence-informed policy revisions.

Policies regarding housing were considered from the perspective that welfare reforms related to housing, as well as rental prices and insecurity of contracts, can be problematic for those vulnerable to food insecurity. One stakeholder discussed how the ‘bedroom tax’ policy meant that people who are vulnerable may have to move, and thereby potentially be required to find a new job, or find themselves without the social support they are accustomed to, such as a nearby family member providing childcare to allow them to work. It was also discussed how rental contracts ending or landlords being able to ask tenants to leave at short notice can cause similar problems, which could result in increased costs, or decreased income, thereby making people more susceptible to food insecurity.

One stakeholder from a political perspective felt that wages need to be addressed by government and rationalised this view with Trussell Trust data showing that most people from their constituency area applying for emergency food packs were working people:

“What is the issue here? It’s low income, because the most people that they see in the food banks and that we see coming in are people with a job.” (Political representative)

Some (*n* = 2) discussed a lack of knowledge surrounding support services for those who are experiencing food insecurity, and an associated need to increase knowledge and access to these services:

“The government can help by making support services maybe better quality, maybe more available, maybe cheaper as well.” (Consumer representative)

Many considered that simply giving people more money by increasing their benefits was not sufficient and that rather providing opportunities to improve skills and education was a needed long-term response to food insecurity. Skills development to decrease vulnerability to food insecurity was considered from three perspectives: (1) the development of employability skills to increase prospective employment and income opportunities, thereby increasing financial access to food, (2) the development of budgeting skills to enable people to use resources more effectively, and (3) developing cooking skills to help people to maximise their budget in relation to food (i.e. enabling people to buy ingredients and prepare meals from scratch rather than choosing more expensive pre-prepared options).

Some stakeholders (*n* = 4) emphasised the importance of sustainability in the agri-food sector and considered how response to food insecurity should be considered alongside implementation of policies to achieve a more sustainable food system.

One discussed sustainability from the perspective of a ‘circular economy’ model, i.e. that NI would be self-sufficient in feeding their own population, rather than the current focus on exporting (particularly the export of meat and dairy products). They considered that this was particularly important as the UK comes out of the European Union, and because of climate change, as consequently in future years the UK may not have as much access to imported food. Others discussed sustainability from an environmental perspective, considering the need for more sustainable practice with regards to food production (particularly meat production) to avoid depleting natural resources, and from the perspective of developing sustainable communities (in terms of promoting local producers and farmers markets, rather than the large multinational supermarkets).

Six stakeholders discussed the issue of ‘holiday hunger’ where children in receipt of school meals during term time may not be adequately nourished during the school holidays due to household food insecurity. Four of these stakeholders cited knowledge of pilot projects or commitments in certain areas to address this issue, indicating that these models could become widespread providing there was government support.

### Opportunities for business response

Although it was acknowledged that several businesses, such as the large multinational retailers, currently are active in responding to the food insecurity cause as part of their CSR strategy and action planning to reduce waste, stakeholders discussed how this should continue, and that more retailers could get involved in providing food to organisations such as Fareshare and food banks:

“The large multinationals, they already do quite a bit to be fair to them on corporate responsibility, but …there’s a lot more supermarkets can do.” (Public Health)

Stakeholders discussed how businesses’ motivation to respond to societal problems such as food insecurity was often strategic to create a positive impression of their organisation. However, one stakeholder from a political perspective considered that this should not be the primary reason, and that businesses should be motivated to invest in society as a moral responsibility:

“Why should we not have a society where business people have social consciences like the rest of us?” (Political representative)

It was considered (*n* = 2) that businesses also have a duty of care to their employees and that supplying food donations to those in need via food banks or other organisations or incentives such as Fareshare may be counterproductive if their own employees are some of those partaking of food charity due to low wages/insecure work contracts:

“In theory you’ve got people working in [supermarket], who may be in poverty…and [supermarket] donates food to the food bank round the corner, and [supermarket] staff go to the food bank to get [food]… They’re going to the food bank, but the food is coming from the place that they work in, so where is the disconnect there?” (Political representative)

## Discussion

The risk factors contained in the model are those which were suggested (with varying levels of consensus, as presented in the preceding section and in the authors previous paper (citation-anonymised for review)) by stakeholders during interviews*.* As evidenced in the literature (Maxwell et al. 2014; Moroda et al. 2018; McKechnie et al. 2018), an individual’s food security status will be determined by the chosen metric for analysis, and can be categorised at different severity levels: food secure, marginal, moderate (low food security), and extreme/severe (very low food security).

Stakeholders discussed various external threats which, aside from household risk factors, can inadvertently contribute to food insecurity. Environmental uncertainty and potential increases in prices of commodities such as fuel and food can increase pressure on the household budget which can further contribute to food insecurity (Lang et al. 2017; Seferedi et al. 2019). Stakeholders discussed how the impending exit of the UK from the EU could further create vulnerability if changes in trade policies and import charges result in cost increases on goods being passed on to consumers. Welfare reforms can contribute to increasing vulnerability for households who receive welfare and rely on this income, and who may find their entitlements being reduced following reforms, or experience waiting times without income while transitioning to the new system (such as the waiting time to transfer to Universal Credit—the streamlined welfare payment system in the UK) (Lambie-Mumford 2014; Loopstra et al. 2018). Although this research was conducted prior to the COVID-19 pandemic, it is reasonable to suggest it to be an additional significant external threat, as the potential related loss or substantial decrease in income for many households may increase their susceptibility to food insecurity (ILO 2020).

Individual level, short-term implications presented in the model (hunger, reduced food choice, inadequate nutrition, worry/anxiety, social exclusion, reduced ability to work/learn, less disposable income) are those which were most prominently discussed by stakeholders and agreed upon in the literature (Alaimo 2005; King et al. 2015; Leonard et al. 2018).

The individual level, long-term outcomes represent individual outcomes resultant from the aforementioned individual level short-term implications. Hunger and inadequate nutrition can cause physical health problems (Kirkpatrick et al. 2010; Moradi et al. 2019), while the anxiety/worry and social exclusion related to not being able to acquire enough food or not being able to participate in social norms such as having friends or family round for food or a drink, or eating out socially, can cause mental health problems (Alaimo 2005; Knowles et al. 2016). Hunger and worry will result in a reduced ability to work and learn which ultimately creates reduced educational attainment and reduced contribution to the workforce (Jyoti et al. 2005; Kruzslicika 2015). Less disposable income results in reduced food choice. Purchases that those on limited budgets make will be higher risk, and this will therefore reduce food choice (Harris et al. 2019). They will be less likely to try new food types or products, and may be less likely to choose healthier options with a lower satiety value than unhealthier options. Purchases can be particularly risky if they have children as they will want to make sure that the foods they buy are those that children will eat, to avoid wasting money. Therefore, low-income consumers are less likely to introduce their children to a wide range of foods which compose a balanced diet, and less likely to persevere with encouraging their children to eat healthy foods such as fruit and vegetables, that they may not at first enjoy (Daniel 2016; Harris et al. 2019). This element of reduced choice and related risk can not only have physical health consequences if it prevents people from making healthy food choices, but further it can also have negative social implications if people feel like they cannot make the same food choices as others, for example if parents are unable to afford to let their children try foods their peers eat.

Individuals experiencing food insecurity who have subsequent poor physical and/or mental health, and less disposable income can produce a collective negative effect on the macro economy and business. Poor health will, in the short-term, decrease contribution to the work force, whether through people being unable to work and therefore being unemployed, or people being employed and taking days off sick, or being present at work but contributing less effectively than they could because of hunger or related health issues (Ramsey et al. 2012; Kruzslicika 2015). Further, poor health resultant from food insecurity/malnutrition represents significant expense for the health service; costs which could be avoided or reduced if national food insecurity levels decreased (Garratt 2017). In addition, those in food insecurity who are unable to work will be reliant on welfare which is an increased cost for the government. Further, hunger or poor health resulting from food insecurity can affect educational attainment, and studies have shown how living in food insecurity can reduce children’s academic progress (Ashiabi 2005; Defeyter 2018). Education generally provides a means for people to earn money and therefore contribute to the economy. Less disposable income reduces the ability of people to contribute to the economy as they will generally have reduced transactions and lower value transactions.

Often those on a lower budget are more concerned with the quantity of food over food quality, choosing foods which are economical and provide greater satiety (Baumann et al. 2017). This desire for ‘cheap’ food may therefore mean that low-income consumers are less likely to contribute to their local economy and instead rely on more affordable supermarkets. This is beneficial for the discounters and large multinational supermarkets, creating opportunity for them to increase market share. However, it is less beneficial for premium priced supermarkets and local stores which may be unable to compete on price. Some stakeholders discussed the reduced choice those on lower incomes have when making food purchasing decisions and how this may make them less likely to choose brands or foods with which they are unfamiliar. Low-income consumers have been found to favour everyday low prices as opposed to promotions (Revoredo-Giha et al. 2018), creating competitive advantage for discounters’, and supermarkets’ own brand product lines. However, the element of risk involved in food purchases for low-income consumers may alternatively result in consumers favouring recognisable, trustworthy market brands, and choosing instead to spend their money on these rather than risk choosing another which they may not be able to consume or replace if they find it unsatisfactory (Baumann et al. 2017). Due to the risk surrounding buying unfamiliar products, retailers could seek to increase trust and reduce risk regarding their own brand ranges via various promotional strategies, such as in-store sampling, a strategy which has been found to be particularly effective in changing lower educated consumers’ purchase behaviour (Heilman et al. 2011).

### Policy implications

Regarding policy/government response, stakeholders discussed a range of responses at both the upstream and downstream levels, as outlined in the conceptual model (Fig. 3). As illustrated in the model, it is hypothesised that policy response across the suggested areas would have a feedback effect on the external threats and household risk factors section of the model, ultimately potentially reducing vulnerability to individual food insecurity. Policy response could also therefore serve to mitigate potential macroeconomic and business implications associated with food insecurity. Stakeholders were in agreement that a food insecurity measure would be a useful first step in the UK to enable further identification of the extent of the problem across time and locations, and to provide an evidence base for decisions pertaining to change, i.e. formation of relevant policies or government recommendations for action and associated funding. Since the stakeholder data collection, it has been announced that food insecurity will be measured in the UK from April 2019 with the first iteration of data publicly available from March 2021 (Butler 2019). This provides evidence of progress in this area, and also that the majority opinion of stakeholders in this study as to the usefulness of a measure is shared by others elsewhere in senior decision-making roles. It will therefore be interesting to observe in the coming years how the resultant data are used by the government to inform solutions to address the problem, as well as being able to monitor the prevalence of food insecurity across the UK and compare across regions using this standardised measure.

Stakeholders further discussed the role of the government in ensuring that people are being fairly paid and provided with necessary skills to adapt to labour market changes. Insecure work contracts (e.g. ‘zero-hour’ contracts) have increased in prominence in recent years (Farina et al. 2020), and have been found to be a significant predictor of household food insecurity (Coleman-Jensen 2011; Purdam and Silver 2020). The uncertainty associated with these contracts has been highlighted during the COVID-19 pandemic with reports of some employee and employer uncertainty with regards to entitlement to government income support (furlough) schemes, and some employer’s reluctance to furlough employees on these contracts (Ogbonna and Franklin 2021). Development of technology and the increasing trend for companies to outsource work has changed the demand for certain skills and the need for certain employees (Trusson and Woods 2017; Martinaitis et al. 2020). Looking towards the future, it is possible that increasing automation may result in those with certain skill sets finding it more difficult to secure employment and therefore increasing their vulnerability to poverty. This triggers debate about the government’s role in preparing for labour market changes, such as investment in skills development and policies to protect low-skilled workers who are most vulnerable (Peyton-Jones 2019). Some sectors in particular have been identified as having employees who are particularly vulnerable to poverty, such as those working in retail, accommodation and food service (Sissons et al. 2018). Therefore, wages in these sectors should perhaps be addressed. Further research on in-work poverty could lead to policy changes around wages and work contracts.

Some stakeholders emphasised the importance of sustainability in the agri-food sector and considered how response to food insecurity should be considered alongside implementation of policies to achieve a more sustainable food system. Although stakeholders differed in the aspect(s) of sustainability they discussed, the need for response was considered from the perspective of all three pillars of sustainability (social, economic, and environmental) (Purvis et al. 2019). The literature also recommends considering food insecurity alongside sustainability, recommending principles related to food justice, resilient local economies, and conservation of natural resources (Nuttman et al. 2020), and the right (and ability) for all in society to financially access healthy food (Elmes 2018). It is also acknowledged however that sustainability and social responsibility must be assessed at all points along the food chain, to ensure that food is not made so affordable for consumers buying it in supermarkets, that there is little profit for producers (Meybeck and Gitz 2017).

At the downstream level, policy frameworks could also recommend implementing local initiatives which could serve to reduce food insecurity in an area; these could include interventions, growing clubs, cooking classes or skills training. Although these were recommended by stakeholders, some also discussed how it was important to actually ask those in food insecurity what initiatives they think would help them, a consideration also cited as important by Furey et al. (2016). Further, although the government provides free school meals to those from disadvantaged backgrounds, they do not provide any food provision to children from these homes during the holidays. There has been an increased focus on ‘holiday hunger’ programmes (Defeyter 2018), therefore perhaps policy should consider financial support for vulnerable families during the holidays. This suggestion has increased validity considering the provisions made by the government during the COVID-19 outbreak to provide children who usually receive free school meals with means to access food (through supermarket vouchers and food from schools’ catering providers) during the mandatory stay at home period (Department for Education 2020).

Food redistribution policies are also of consideration, as mutually beneficial partnerships between food production/retailing/service businesses and organisations such as Fareshare are increasing. As businesses can have reservations about redistributing food for reasons of cost or reputation (Alexander and Amaje 2008; Sert et al. 2018), policies which incentivise or facilitate operations for businesses to redistribute food could be useful. Policies such as this have already been implemented in Europe (Italy and France) and North America. For example, in France, tax breaks and fines for noncompliance are used to incentivise supermarkets to redistribute surplus food (Cohen 2021). As acknowledged by stakeholders, several retailers and other food businesses such as manufacturers are already actively helping the food insecurity agenda with various activities such as food redistribution and donations, which are often aligned to CSR strategies. Activity relating to food redistribution and donations should be maintained and increased as appropriate, and there is scope for new food businesses to partner with organisations to further support the food insecurity agenda. Some stakeholders discussed how having a duty of care to their employees should encourage businesses to get involved in addressing food insecurity. Further, particularly as some sectors have been identified as low-paid, it is important to ensure employees are fairly treated to avoid a disconnect where the employees of an organisation donating to food banks are visiting said food banks as they are receiving inadequate pay (Rayner 2019). As illustrated in the model, business response to address food insecurity can potentially feedback to reduce risk to household food insecurity (e.g. by increasing access to food), or can help to mitigate circumstances in the short term (e.g. through surplus food donations to food banks).

Although stakeholders discussed various areas that could be addressed by government to improve the problem, it was generally acknowledged that addressing one singular issue in silo would not be sufficient and that instead a co-ordinated long-term approach was needed, thereby justifying the need for a government strategy. The importance of a cross sectoral, and cross-departmental, collaborative response was emphasised to provide stronger solutions and save resources, rather than different sectors and departments working on the same problem independently. Murray, Haynes and Hudson (2010) agree that it is unlikely that solutions to societal problems will be found in any one department or organisation and that collaboration would therefore be useful to support measures for a more responsible, sustainable economy. King et al. (2015) also discuss the importance of a collaborative approach regarding food insecurity response, and state that any response should involve creation of a common agenda for organisations to work towards and linked practicable actions. Overall, stakeholders however felt that the cost involved would be a barrier to government acting to implement policy addressing food insecurity, and that a strong case as to the long-term economic benefits of its reduction was therefore necessary.

### Limitations

A limitation of this research was the absence of getting input from food insecurity ‘experts by experience’. However, the ability to get insights from those who have experience working with those in food insecurity on a daily basis, and who are aware of their experiences enabled a wider reach of perspectives than would have been possible had a similar number of those experiencing food insecurity been interviewed. Further, previous research has examined in depth individual implications of food insecurity, whereas the objective of this study was concerned with how these individual implications impact on business and the economy, and what types of business and governmental response would be useful and/or feasible. Therefore the sample was appropriate in containing people whose work remit considers these issues as they could provide a more informed response from this perspective.

## Conclusion

The conceptual model emanating from this research provides a contribution to the theoretical literature in this field by providing a schematic overview of the relationship between household food insecurity, individual implications, macroeconomic implications, and opportunities for business and policy to respond. It is widely acknowledged in the literature that food insecurity has numerous individual implications (e.g. implications for both physical and mental health and wellbeing). This model acknowledges these individual implications and further considers the potential ultimate impact of food insecurity on the economy (e.g. reduced contribution to the local economy, increased cost burden for the National Health Service). This model can therefore inform and rationalise business and policymakers’ actions to respond to the issue of food insecurity, for example corporate social responsibility initiatives related to food insecurity, and targeted policy response related to problem areas suggested (e.g. wages and work contracts). The model can further provide researchers with areas for future research and debate, for example quantitative investigation of some of the elements included in the model, or further qualitative study with those experiencing food insecurity, or with stakeholders in other geographical locations.

## Data Availability

The data from this study are not publicly available due to privacy and ethical constraints.
